# Regulated and unregulated emissions from Euro VI Diesel and CNG heavy-duty vehicles

**DOI:** 10.1016/j.trd.2024.104349

**Published:** 2024-09

**Authors:** Roberto Gioria, Tommaso Selleri, Barouch Giechaskiel, Jacopo Franzetti, Christian Ferrarese, Anastasios Melas, Fabrizio Forloni, Ricardo Suarez-Bertoa, Adolfo Perujo

**Affiliations:** a European Commission, Joint Research Centre (JRC), 21027 Ispra, Italy; b European Environment Agency (EEA), 1050 Copenhagen, Denmark; c ETSI Minas y Energía, Universidad Politécnica de Madrid, Paseo Juan XXIII 11, Madrid, Spain

**Keywords:** Real world emissions, PEMS, Non-regulated pollutant, Euro 7, SPN, GHG

## Abstract

This study compares emissions from Euro VI-D Diesel and CNG buses across temperatures from −7 °C to 35 °C. Pollutants including NOx, THC, CH_4_, CO, NH_3_, N_2_O, HCHO, Solid Particle Number larger than 23 nm (SPN23) and larger than 10 nm (SPN10) were measured. Both buses complied with Euro VI-D but exceeded European Commission’s proposed Euro 7 limits, notably for NOx and SPN10. The CNG bus also surpassed NH_3_, CO, and CH_4_ limits, while the Diesel exceeded N_2_O limits. High NH_3_ emissions were observed from CNG (up to 0.320 g/kWh), with Diesel reporting lower levels (up to 0.021 g/kWh). HCHO emission from both vehicles were very low. SPN23 was under limits, but SPN10 exceeded Euro 7 limits at cold start tests. CNG’s CH_4_ and N_2_O emissions constituted up to 4.6% and 3.5% of CO_2_ equivalent, respectively. Diesel bus showed negligible CH_4_ but N_2_O emissions represented up to 37% of CO_2_ equivalent.

## 1 Introduction

Air pollution is a major environmental health risk in Europe, particularly in urban areas (WHO, 2023), with the European Environment Agency (EEA) reporting that over 200,000 premature deaths in the EU27 could be related to air pollution (European Environment Agency, 2020). Worldwide, the annual number of premature deaths due to air pollution reaches 4.2 million, with low- and middle-income countries bearing 89% of this burden. While emissions of key air pollutants have decreased in Europe over the past two decades, air quality remains a concern in various areas (WHO, 2023). The electrification of some road transport modes is expected to reduce air pollution, but for others reliant on fuel combustion, even if carbon neutral, air pollution will continue to be a significant concern. Consequently, the improvement of urban air quality has motivated the implementation of various European policies, initiatives, and actions.

Road transport significantly contributes to pollutants that impact air quality, with road traffic accounting for approximately 11% of particulate matter (PM), 28% of black carbon, and 37% of nitrogen oxides (NOx) concentrations in Europe (European Environment Agency, 2020). To address this, the European Union has implemented increasingly stringent vehicle emissions standards. Despite Heavy-Duty Vehicles (HDVs), such as buses, represent a small percentage of the total vehicle population (4% of the on-road fleet in the European Union (Muncrief and Sharpe, 2015), they are responsible for approximately 27% of on-road carbon dioxide emissions (CO_2_). Additionally, HDVs have been shown to be responsible for 40–60% of PM and NOx emissions from road transport (European Agency, 2020b).

HDVs encompass a wide and significant range of vehicles in the road transport sector, serving various functions such as transporting passengers and freight, as well as fulfilling specialized applications. This results in a wide range of HDV characteristics, types, and uses.

The type approval of a HDV is based on the engine and is carried out on a test dynamometer using a standardized test cycle where the engine speed and torque are varied. The same engine can be utilized for various purposes, for example, in a truck (category N) or a bus (category M). The Euro VI legislation (Regulation (EC) No 582/2011 (2011)) includes a test using Portable Emissions Measurement System (PEMS) as part of the type approval process, and subsequent In-Service Conformity (ISC) testing. ISC is designed to verify that the engine's emissions remain below regulated limits throughout its useful life without requiring the engine to be extracted from the vehicle (Regulation (EC) No 595/2009 of the European Parliament and of the Council of 18, June 2009). The PEMS test is conducted in real-world driving conditions, requiring trips to meet practical limitations including operation shares, route composition, and engine workload. The same emission limits apply for both Diesel and Compressed Natural Gas (CNG) engines/vehicles, except for hydrocarbon emissions (THC), which are split into non-methane hydrocarbons (NMHC) and methane (CH_4_) for CNG.

In recent years, several cities around the world, such as New Delhi, Beijing, and Madrid, have adopted CNG technologies to update their city bus fleets (Yue et al., 2016, EMT-Madrid Flota autobuses, 2020). Advocates of CNG technologies argue that they offer advantages such as the potential to reduce NOx by using a stoichiometric engine with a Three-Way Catalyst (TWC), as well as achieving lower PM emissions due to the chemical composition of the fuel, which is mainly CH_4_ (Bielaczyc et al., 2015, Karavalakis et al., 2013, Prati et al., 2022, Zhu et al., 2020). However, CNG technologies have been associated with high Solid Particle Number larger than 10 nm  (SPN10) (Giechaskiel, 2018, Lähde, 2022, Kontses et al., 2020) and ammonia (NH_3_) emissions (Suarez-Bertoa et al., 2020, Thiruvengadam et al., 1995, Vojtíšek-Lom et al., 2018).

On the other hand, Diesel engines have become progressively cleaner, particularly due to their after-treatment systems, which have been encouraged by the application of a more stringent regulatory protocol (Euro VI − Step D and E). Today’s Euro VI heavy-duty vehicles include Diesel particulate filters (DPF) and Selective Catalytic Reduction (SCR) systems that can reduce particulate and NOx emissions (Grigoratos et al., 2019, Selleri et al., 2021). However, an increase in nitrous oxide (N_2_O) emissions has been reported for vehicles using SCR systems (Selleri et al., 2021, Vojtíšek-Lom et al., 2018). Given HDVs significant impact on the overall greenhouse gas (GHG) emissions and air pollution, emissions of compounds such as CH_4_, N_2_O, formaldehyde (HCHO), and SPN10 need to be carefully evaluated. This has been partially reflected in the recent Euro 7 European Commission proposal.

This study aims to investigate various aspects related to emissions from buses fueled by Diesel and CNG. The work compared two buses of recent generation, both from the same manufacturer and having similar characteristics in terms of power and engine displacement. The evaluation was conducted using the Euro VI D methodology, as well as methods included in the Euro 7 proposal made by the European Commission. The comparison provides insights into the emissions of these buses and contribute to understanding the environmental effects of different fuel types and technologies in the context of urban air quality and greenhouse gas emissions.

## 2 Materials and methods

The emissions of a Diesel Engine coach bus (M3 Class III) and an urban transit bus powered by CNG (M3 Class I), that met the Euro VI Step D standard, were investigated on the road during real-world conditions, and in the laboratory, covering a various range of ambient and system conditions (see section 2.2.2.). NOx, THC, CH_4_, CO, NH_3_, N_2_O, HCHO, Solid Particle Number larger than 23 nm (SPN23) were measured over on-road and laboratory tests, with the exception of HCHO and SPN10 that were only measured in the laboratory.

### 2.1 Tested vehicles

The two buses belong to category M3, as defined in the Annex II of Directive 2007/46/EC. The vehicles and engines specifications are summarized in Table 1 (vehicles) and Table 2 (engines).

**Table 1:** Vehicles details and specifications.

	HD Diesel	HD CNG
Vehicle category	M3 Class III	M3 Class I
Emission standard	Euro VI Step D	Euro VI Step D
Fuel type	Diesel	CNG
Length (mm)	12,097	12,050
Vehicle mass in running order (kg)	12,550	12,520
Vehicle technically permissible max laden mass (kg)	19,300	19,500
Axle layout	2 axis-4 wheels-6 tires	2 axis-4 wheels-6 tires
Transmission/Gear box	Automatic	Automatic
Max Speed (km/h)	100	85

**Table 2:** Engine details and specifications.

	HD Diesel	HD CNG
Working principle	Compression ignition	Spark ignition
Engine configuration	6 cylinders in line	6 cylinders in line
Engine size (cm^3^)	8710	8710
Maximum power	265 kW @ 2200 rpm	264 kW @ 2000 rpm
Maximum torque	1650 Nm @ 1200 rpm	1640 Nm @ 1200 rpm
After-treatment configuration	DOC+DPF+SCR/ASC	TWC

### 2.2 Tests performed

#### 2.2.1 On-road tests

For each bus, four routes were specifically designed. The first route, known as ISC, follows the requirements of Regulation (EU) 582/2011 and its amending act (Regulation (EC) No 2016/1718 (2016)). This test aims to indirectly assess the engine's conformity to the type-approval limit values. The route comprises varying shares of urban, rural, and motorway sections, tailored according to the vehicle class, and was different for the two buses.

**Fig. 1:**
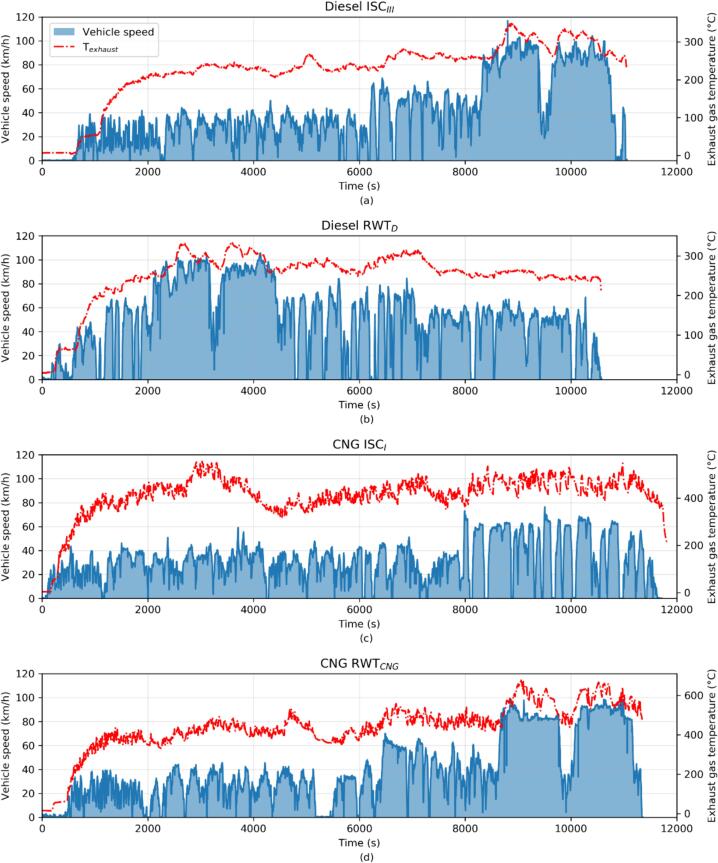
Real-world routes speed profiles (blue area) and the exhaust temperature for Diesel ISC_III_ (a) and RWT_D_ (b), and for CNG ISC_I_ and RWT_CNG_ test routes. (For interpretation of the references to colour in this figure legend, the reader is referred to the web version of this article.)

Real-World Test (RWT), the second route in real-world conditions, includes different shares of urban, rural, and motorway segments compared to the ISC. It was created to examine the impact of trip shares. To emulate real-life usage, the Diesel bus underwent a test without specific limitations on the urban, rural and motorway composition, and the CNG bus was tested not only over urban and rural operation but also over motorway on a trip with shares similar to the Diesel ISC route. Additional details of each cycle and route are presented in Table 3, and illustrated in Fig. 1.

**Table 3:** Road tests matrix. All tests started with engine at ambient temperature (i.e. cold start). The payload was 43 % for all tests for both vehicles.

	Test Cycle	Ambient Temperature (°C)	Total Work (kWh)	Duration (minutes)	Shares U/R/MW (%)
**Diesel**	ISC_III_	5 ÷ 7	121	176	50/22/28
	RWT_D_	5 ÷ 7	136	180	44/36/20
**CNG**	RWT_CNG_	4 ÷ 5	119	181	49/24/27
	ISC_I_	4 ÷ 5	109	195	69/31/0*

*Data processed using speed bin method. 3/9/88 (U/R/MW) using the first acceleration method.

**Fig. 2:**
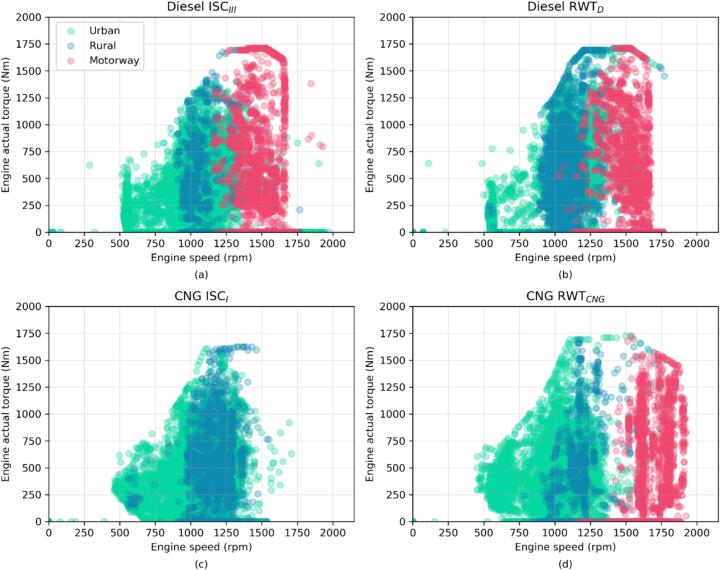
Engine mapping with the sub-session division (“Urban” in green, “Rural” in blue and “Motorway” in red) for Diesel ISC_III_ (a) and RWT_D_ (b), and for CNG ISC_I_ and RWT_CNG_ test routes. Note that the “Motorway” part is not present in the ISC_I_ for the CNG vehicle. (For interpretation of the references to colour in this figure legend, the reader is referred to the web version of this article.)

Fig. 1 illustrates vehicle speed, exhaust temperatures for all the routes. In Fig. 2 engine mapping divided into the considered speed bins (U/R/M) is displayed. The payload percentage, set at 43%, has been selected to compare the vehicles under the same load.

The measurement setup utilized during the road tests is summarized in Figure S1 of the Supplementary Material. Further details on the instrumentation used in the lab are described in the measurement equipment section. Table S1 (see Supplementary Material) depicts additional details on the statistics of the routes used to perform road tests. Figure S2, in the Supplementary Material, depicts the trip segmentation as required by Regulation (EU) 2016/1718 (Regulation (EC) No 2016/1718 (2016)) for the two different vehicle categories.

#### 2.2.2 Laboratory tests

The evaluation of emissions from the two buses was conducted using the World Harmonized Vehicle Cycle (WHVC) and In-Service Conformity-like tests on a chassis dynamo across a wide ambient temperature range, ranging from −7 °C to 35 °C, including intermediate points at 0 °C and 22 °C. The WHVC is designed to replicate the Type Approval (TA) test conducted on engines during the homologation phase, known as the World Harmonized Transient Cycle (WHTC). It is important to note that WHVC is not a regulated cycle, but it has been shown to be an effective proxy for transposing the TA test from the engine to the roller dynamometer, as both cycles were built based on the same database (Regulation (UN/ECE) No 49 (2010); GTR No. 4 (UNECE), 2007. Table 4 provides a list of the laboratory tests performed during the campaign and summarizes the main features of the tests, including the test cycle, coolant condition, payload, and ambient temperature. A 43% payload, the same as for the road test, was used to achieve a work over the WHVC similar to the engine value over the WHTC, which serves as a reference point for the tests.

**Table 4:** Laboratory test matrix.

	Test Cycle	Engine at Start	Payload (%)	Ambient Temperature (°C)
**Diesel**	WHVC	Cold + Hot	43	35, 22, 0, −7
	ISC_III_-like	Cold	43	35
**CNG**	WHVC	Cold + Hot	43	35, 22, 0, −7
	ISC_I_-like	Cold	43	35

The WHVC tests were conducted in both cold and hot starting conditions and then weighted using the methodology specified in Regulation (EU) 582/2011 and 2016/1718 for engines over the WHTC. In this case, the cold portion, i.e., test starting at a coolant temperature below or equal to 30 °C, has a weight factor of 14%, and the hot portion, i.e., part of the test starting at a coolant temperature above 70 °C has a weight factor of 86%. The final test result is hence a weighted average of the cold start test and the hot start test according to the following equation (Regulation (UNECE), No 49, 2010:

(1) $$e=\frac{(0.14\;\times \;m_{\mathrm{cold}})\;+\;(0.86\;\times \;m_{\mathrm{hot}})\;}{(0.14\;\times \;W_{\mathrm{act,cold}})\;+\;(0.86\;\times \;W_{\mathrm{act,hot}})\;}$$

where:

m_cold_ = is the mass emission of the component on the cold start test, g/test.

m_hot_ = is the mass emission of the component on the hot start test, g/test.

W_act,cold_ = is the actual cycle work on the cold start test, kWh.

W_act,hot_ = is the actual cycle work on the hot start test, kWh.

Before the “cold” tests, an overnight soaking period was allowed to ensure that the main relevant parts of the vehicle, such as the engine, after treatment devices, and fuel (including Diesel Exhaust Fluid (DEF) where applicable), reached the ambient temperature. In all cases, the vehicle's coolant and oil were within ± 3 °C of the set point ambient temperature. Additionally, In-Service tests (ISC_III_-like and ISC_I_-like tests) were also conducted in the laboratory, closely replicating the road test in terms of speed and the road slopes profile followed by the vehicle. To properly simulate the slope of the road, the road loads and the road gradient were calculated, resulting in a virtual path comparable with the real-world one. Figure S3, in the Supplementary Material, describes the vehicle speed profiles used during laboratory tests.

### 2.3 Measurement setup

The laboratory tests were conducted at the Heavy-Duty Vehicle Laboratory of the European Commission’s Joint Research Center (EC JRC), located in Ispra (Italy). This facility comprises a climatic test cell capable of operating within a temperature range of −30 °C to 50 °C, with controlled humidity. It is equipped with a 2-axis roller dynamometer, 4 wheel-drive (4WD), specifically designed for Heavy-Duty vehicles.

Figure S4, in the Supplementary Material, represents the testing layout. A dilution tunnel utilizing constant volumetric flow (CVS) was employed to dilute the vehicle exhaust. The CVS flow rate was 100 m^3^/min achieving a dilution ratio of at least 6:1 at the highest engine loads (i.e. max exhaust flow rate). The transfer line was 9 m long.

#### 2.3.1 Laboratory analysers

The measurement of criteria pollutants from the vehicle's tailpipe was carried out using an AVL AMA i60 analyser (AVL Ama i60., 2019), which was connected to the tailpipe to measure hydrocarbons (THC) using a flame ionization detector (FID), CO and CO_2_ with a non-dispersive infrared (NDIR) analyser, and nitrogen oxides (NOx) using a chemiluminescence detector (CLD). Additionally, a Fourier-Transform Infrared Spectroscopy (FTIR) analyzer was used to measure tailpipe emissions of NH_3_, N_2_O, and HCHO (AVL FTIR SESAM, 2019, Gioria et al., 2020).

The measurement of SPN23 was carried out from the dilution tunnel using an AVL particle counter (AVL APC, 2019). The APC consisted of a hot dilution stage, an evaporation tube, a cold dilution stage, and a condensation particle counter (CPC) with a 50% cut-off size at 23 nm. Additionally, SPN10 was measured using a TSI 3792 CPC connected to the outlet of the APC’s cold dilution stage. The overall efficiency at 10 nm was around 30% due to losses in the APC (Giechaskiel and Melas, 2022).

#### 2.3.2 Portable measurement analysers

During the on-road tests three different portable measurement instruments were used, an AVL MOVE PEMS that measured the criteria pollutants and CO_2_ from both vehicles, a PEMS-LAB, produced by CERTAM-ADDAIR, was installed on the Diesel vehicle and an HORIBA OBS-ONE-XL was used on the CNG vehicle. These two were on-board to measure N_2_O and NH_3_ from the vehicles’ tailpipe.

The AVL MOVE PEMS (AVL Move PEMS, 2019) was used to measure the exhaust gas concentrations, with CO and CO_2_ measured with a NDIR, CH_4_ and THC using a FID, and NOx with a non-dispersive ultra-violet sensor (NDUV). The PEMS system included heated sampling lines at the tailpipe, an exhaust flow meter (EFM), a data logger connected to the OBD port of the vehicle, a weather station for recording ambient temperature and humidity, and a GPS for calculating road grades. The system recorded emissions at a frequency of 10 Hz and then performed moving averaging and resampling to 1 Hz. Access to the vehicle’s Engine Control Unit (ECU) provided real-time engine status and essential parameters for determining engine work, as well as auxiliary signals including vehicle speed, coolant temperature, torque, and engine speed.

The PEMS-LAB (Amanatidis et al., 2016) is equipped with a portable FTIR analyser (hereinafter p-FTIR) and employs a PTFE tube to directly sample exhaust from the vehicle's tailpipe. In order to avoid condensation and/or adsorption of hydrophilic compounds such as NH_3_, the line is heated to 220 °C.

HORIBA’s OBS-ONE-XL applies Infrared Laser Absorption Modulation (IRLAM) technique, which relies on infrared absorption spectroscopy and employs a Quantum Cascade Laser (QCL) as light source (Onishi et al., 2021, Selleri et al., 2022, Shibuya et al., 2021). The sampling line, which extends to 6 m, is equipped with a PTFE and an in-line filter. It is heated to 113 °C to guarantee a quick response time, minimize adsorption, and avoid condensation. Additional information can be found in Suarez-Bertoa et al. (2022).

### 2.4 Evaluation and data processing methodology

Two methodologies were used for the data evaluation. The experimental data were analyzed using both the requirements prescribed (i) by the regulatory homologation process for the specific vehicles, i.e., Euro VI Step D and (ii) following the approach included in the European Commission’s Euro 7 proposal (Proposal for a regulation of the European Parliament and of the Council on type-approval of motor vehicles and engines and of systems, 2009, Thedinga et al., 2023).

For the laboratory tests, the exhaust flow was determined by subtracting the dilution air flow to the total CVS flow. For the on-road tests, the mass emissions at the tailpipe were calculated using the exhaust flow measured by the EFM. Additionally, the work was determined using the torque and the engine speed signals provided by the ECU.

#### 2.4.1 Euro VI Step D data analysis

Since both vehicles were Euro VI Step D buses, the ISC tests were analyzed according to the Regulation (EU) 2016/1718 (Regulation (EC) No 2016/1718 (2016)). The procedure unsed in the analysis of the data is based on calculating the mass emissions for every partially overlapping subset of the complete data set, also called window.

The size of each window is equal to the work measured over the reference laboratory transient cycle. The window had roughly a specific length of 25.48 and 25.30 kWh, respectively, for the Diesel and CNG buses. The first window is generated at t = 0, i.e. at the beginning of the test. A new window is generated after every second. The pollutant emissions are then integrated in each window. Emission factors are obtained by dividing the integrated emissions by the window’s work. After establishing the windows, the 90^th^ percentile of the cumulative windows distribution were calculated. A set of boundary conditions must be respected to determine the emission and obtained the valid windows used in the analysis (Regulation (EC) No 582/2011 (2011)). The most important is that cold start data are not considered for the Euro VI Step D. The analysis starts when the coolant temperature reaches 70 °C for the first time or stabilizes within +/– 2 °C over 5 min, whichever happens first, but not exceeding 15 min after engine start. The time taken to reach a coolant temperature of 70 °C must be under urban driving conditions. Following the cold start, the share of urban, rural, and motorway driving is determined. The calculations were performed using EMROAD V 6.05 B3. (Bonnel, 2015, EMROAD), a Microsoft Excel add-in used to analyze on-road emissions data collected with PEMS

The emissions over the WHVC at 22 °C were calculated weighting the emissions resulting from the cold and hot WHVC tests, using the definition laydown in the UNECE Regulation 49 for engine testing over the WHTC (see equation (1)). This was used as a proxy for the WHTC emissions.

#### 2.4.2 Euro 7 proposal data analysis

The data have been also evaluated with the approach included in the Commission Proposal for the Euro 7 legislation (Proposal for a regulation (EC) No. 586 (2022)). The tests duration must have a total work over 3 times the reference work of the Type Approval engine cycle WHTC. Emission factors from the on-road and the ISC-like tests were computed using moving windows with a length equal to the reference work. The windows start from the beginning of the test with time increments of 1 s, similarly to what was used in the Euro VI D analysis. After determining all the windows, the 100^th^ and 90^th^ percentiles of the cumulative windows distribution were calculated.

Emissions during the WHVC tests were calculated as total emissions of each pollutant over the cycle were divided by the worked performed. The emissions obtained from WHVC_Cold_ were compared to the “Cold limit” and the emissions from WHVC_Hot_ were compared to the “Hot limit”. Note that the Cold limit and Hot limit correspond to the limits set for 100^th^ and 90^th^ percentiles of the cumulative windows in the on-road tests, respectively. EU emission limits for each pollutant in EURO VI and those proposed by the EC for the Euro 7 are shown in Table 5.

**Table 5:** EU emissions limits (g/kWh, SPN in #/kWh) for heavy-duty engines and vehicles.

Emission Standard	NOx	CO	THC	NMHC	CH_4_	NH_3_*	NMOG	PM	SPN23	SPN10	N_2_O	HCHO
Euro VI Diesel	0.460	4.0	0.160	−	−	10	−	0.010	6.0x10^11^	−	−	−
Euro VI CNG	0.460	4.0	−	0.160	0.50	10	−	0.010	6.0x10^11^	−	−	−
Euro 7** −100^th^perc./Cold emissions	0.350	3.50	−	−	0.50	0.065	0.20	0.012	−	5.0x10^11^	0.160	0.030
Euro 7**- 90^th^perc./Hot emissions	0.090	0.20		−	0.350	0.065	0.050	0.008	−	2.0x10^11^	0.10	0.030

*In Euro VI a limit for NH_3_ (in ppm) is set for the engine test over the WHTC; **Euro 7 limits apply to all fuel and engine technologies.

## 3 Results and discussion

This section presents the exhaust emissions of NOx, THC, CH_4_, CO, NH_3_, N_2_O and SPN23 that were measured on-road and on a chassis dyno over the WHVC at different ambient temperatures. In addition, in the laboratory also HCHO and SPN10 exhaust emissions were measured. The analysis was done using the Euro VI Step D and Euro 7 evaluation methods described above.

### 3.1 Euro VI Step D evaluation

The vehicles environmental performance was investigated according to the Regulatory framework at which they were type approved, i.e., the Euro VI Step D (Regulation (EC) No 2016/1718 (2016)). The emissions according to the Euro VI Step D are summarized in Table 6. SPN and average concentration of NH_3_ are not regulated during on-road ISC in Euro VI Step D, but are reported for completeness.

**Table 6:** Emission factors of the 90^th^ percentile window during the corresponding ISC test and the weighted emissions over the WHVC at 22 °C performed with the Diesel M3 Class III HDV (ISC_III_) and CNG M3 Class I HDV (ISC_I_). Note that occasionally CH_4_ values can be higher or very close to THC ones, because the emissions were close to the instrument's detection limit. The emissions of the two vehicles were below the Euro VI limits (see Table 5).

Bus by fuel type	Test Cycle	CO_2_ (g/kWh)	CO (g/kWh)	NOx (g/kWh)	THC (g/kWh)	CH_4_ (g/kWh)	NH_3_ (ppm)	SPN23 (#/kWh)
Diesel	ISC_III_	771.7	0.227	0.393	0.011	0.011	1	NA
	WHVC	856.8	0.137	0.396	0.004	0.001	2	1.9E+11
CNG	ISC_I_	809.8	0.388	0.246	0.008	0.010	13	8.3E+09
	WHVC	874.5	0.613	0.323	0.072	NA	2	1.7E+10

During both the on-road and laboratory tests, the emissions of the two vehicles were below the Euro VI limits. While presenting comparable CO_2_ emissions (CNG higher 2–5%), the CNG vehicle had higher CO emissions compared to the Diesel vehicle, but still within 10–15% of the limit. The NOx emissions from the Diesel vehicle were slightly higher than those from the CNG’s and at 86% of the limit (without considering any conformity factors). The Diesel's SNP23 was one order of magnitude higher than the CNG's (at a level around 30% from the limit).

**Fig. 3:**
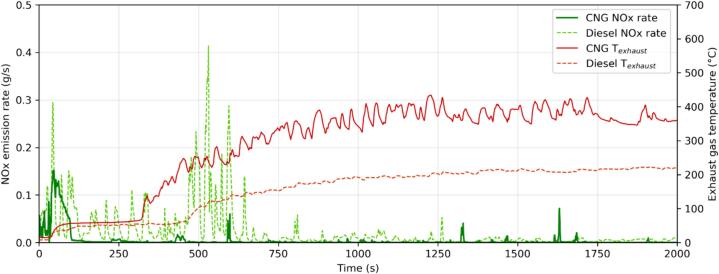
Comparison between an ISC test for Diesel (ISC_III_) and CNG (ISC_I_) buses. Exhaust gas temperature in red, NOx instantaneous emissions in green (Diesel: dashed lines: CNG: solid lines) The data were aligned using the engine starting point as the main event. (For interpretation of the references to colour in this figure legend, the reader is referred to the web version of this article.)

As expected, the Diesel presented low emissions of THC, which, for both vehicles, were mainly composed of CH_4_. The CNG presented low THC and CH_4_ emission factors during the road test, and higher during the laboratory testing, but still lower than half of the limit (applicable only to the Diesel). THC emissions from Diesel vehicles, including CH_4_, are known to be low, and little affected by cold start, because of the good efficiency of the combustion process and that of the DOC (Xin, 2016). CH_4_ emissions from the CNG can be associated to incomplete combustion of the fuel, which comprised mainly methane, and the difficulty of the TWC to oxidize this molecule (Ferri et al., 2018). The same trend as CH_4_ was also present for other pollutants, e.g., CO and SPN23, and was linked to the contribution of the cold start emissions that are excluded from the on-road ISC Euro VI Step D analysis, affecting the final emission factor. As illustrated in Fig. 3, the CNG vehicle presented a large amount of the NOx emissions, most of which occur during cold start, or at least until the TWC reached the light-off temperature, namely after about 2 min.

Fig. 3 illustrates the real-time NOx signals and temperature profiles for the two buses. NOx emissions from the Diesel bus were elevated during the first 650 s. Most likely, this has to do with the limited DEF injection below 200 °C (Bielaczyc et al., 2015, Guo et al., 2015, Hallquist et al., 2013, Jiang et al., 2018). The CNG bus, with TWC, has high NOx emissions only during the first 60–120 s, the time for the TWC to reach the light-off temperature. Note that, since the SCR and TWC temperatures were not available, the exhaust gas temperatures were used as proxy.

Several factors contribute to the determination of emission levels, including ambient temperature (Bermúdez et al., 2015), engine combustion temperature (which is linked to the air–fuel ratio) (Mera et al., 2019), after-treatment system capacity (Smit et al., 2019), among others. The urban contribution is predominant for both vehicles, as reported also by other authors (Mendoza-Villafuerte et al., 2017, Söderena et al., 2018), because of the lower efficiency of the aftertreatment devices. In addition, while the efficiency of the SCR is influenced by the operating temperature, with the TWC, it seems more important to have an adequate fuel-to-air ratio, as any variation can largely affect the conversion efficiency of NOx. The Supplementary Material gives examples of exhaust gas temperatures profiles during the tests (see Figure S5 in the Supplementary Material), as well as a direct correlation between exhaust gas temperature and NOx emission rate for Diesel (SCR system) and CNG (TWC system) buses at different route. Coincidentally, the Diesel’s NOx emissions are in excellent agreement with the best performing of two HD vehicles reported by Zhu et al. (∼0.34 g/kWh) for vehicles certified at 0.2 g/bhp-hr level (Zhu, et al., 2024). Interestingly, the data analysis required by the US EPA includes cold start emissions (completely excluded by the Euro VI D). This could suggest that the vehicle presented lower emissions than those measured here or that integrating the emissions over the entire test (with or without binning) dilutes the contribution of the cold start and urban driving over the entire test. Moreover, the same study reports one order of magnitude lower NOx for two CNG vehicles. The CO levels of the CNG vehicles reported by Zhu et al. are at least 3.7 times higher than those reported in the present study. This shows again the importance of the fuel-to-air ratio strategy for TWC equipped vehicles, that in Zhu et al. appeared to run rich. CO formation in an engine is governed by the fuel-to-air ratio, being favored by stoichiometric or slightly rich mixing conditions, along with relatively short residence times in the combustion chamber, where oxidation reactions are incompleted (Zhu et al., 2020). Such conditions, which are typically encountered in a spark ignition engine as the CNG bus engine, could cause large CO emissions. The thin equilibrium, tilting toward rich combustion, favors lower NOx at expenses of emitting more CO. This is in line with most studies under controlled laboratory conditions, which have indicated that CO emissions from CNG buses are higher compared to those from Diesel (Giechaskiel et al., 2019, Selleri et al., 2021), due to the fact that stoichiometric CNG engines operate at a richer air–fuel-ratio (λ ≤ 1) than Diesel engines (Järvinen, et al., 2019). This strategy likely results from the more stringent limit on NOx compared to CO.

### 3.2 Euro 7 proposal evaluation

The present section analyses the behavior of the two vehicles using the methodology included in the European Commission’s proposal for the future Euro 7 regulation.

**Fig. 4:**
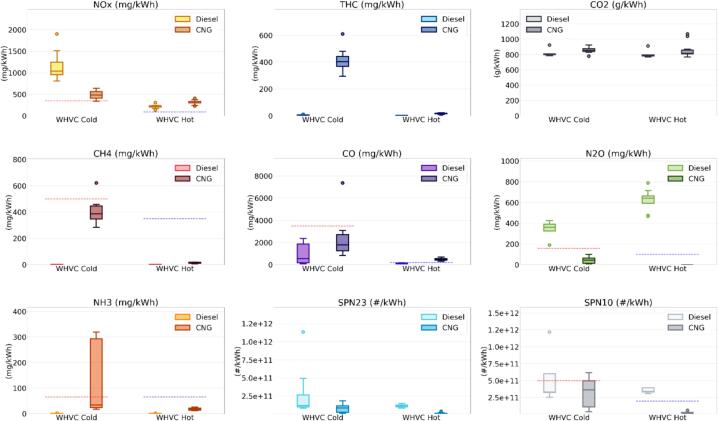
Overall emissions of the WHVC_Cold_ and WHVC_Hot_ tests performed with the Diesel and CNG buses at ambient temperatures ranging from −7 to 35 °C. No correction has been applied for any of the instruments used. Box plot are Q1-Q3 interquartiles, horizontal line Q2. The whiskers extend to 1.5 times the Q3-Q1 difference. Dots indicate outliers. Dotted red and blue lines indicate the Euro 7 limits, as laid down in the European Commissions’ proposal for WHVC_Cold_ and WHVC_Hot_ tests, respectively. (For interpretation of the references to colour in this figure legend, the reader is referred to the web version of this article.)

Fig. 4, depicts the aggregate results of all the WHVC_Cold_ and WHVC_Hot_ experiments performed at different ambient temperatures, −7, 0, 22 and 35 °C. A summary with the emission factors can be found in Table S2 of the SM. It should be noted that Fig. 4 provides a general overview of the vehicles’ emission performance without claiming to be statistically representative, due to the limited number of tests and the numerous effects taken into account. It should be considered as an indication of the variability in emissions within broadly defined operative conditions boundaries. Furthermore, it is important to highlight that the tests conducted did not cover all the potential usage scenarios. The median emissions from the two buses (see Table S2 of the SM) show that both vehicles would comply with the Cold and Hot limits proposed for HCHO, CH_4_ and NH_3_. The emissions of HCHO were very low (<0.001 g/kWh) for both vehicles during all WHVC tests. The highest emissions, 0.002 g/kWh, were registered from the CNG vehicle at −7 °C. The emissions were in line with previous studies (Lähde and Giechaskiel, 2021, Suarez-Bertoa et al., 2021).

Although below the limits, the CNG presented substantially higher median emissions of CH_4_ and NH_3_ (0.388 g/kWh and 0.034 g/kWh for CH_4_ and NH_3_, respectively) during the WHVC_Cold_ tests as compared to the Diesel, for which the emissions were very low (less than 0.002 g/kWh), as expected. The CNG’s median emissions of CH_4_, a short-lived greenhouse gas (atmospheric lifetime of 12 years) with a global warming potential (GWP) over 20-year timeframe 84 – 87 times higher than CO_2_, were equivalent to 33 gCO_2_eq/kWh or 3.8% of the CO_2_ emitted by the vehicle. The CNG emissions decreased during the hot operation but NH_3_, with 18 mg/kWh, remained far from negligible and higher than the Diesel.

NH_3_ is an important precursor of PM2.5 and plays an important role in the eutrophication of waters (Suarez-Bertoa et al., 2019, Pusede, 2015). Different pathways lead to the emission of NH_3_ from the technologies studied. On one hand, the NH_3_ emissions from spark ignition engines equipped with TWC are related to fuel enrichment after the light-off of the TWC (Bouwman et al., 2002, Heeb et al., 2008). During fuel rich events, H_2_ is formed from the reforming of hydrocarbons, which by reacting with NOx over the TWC forms NH_3_. Indeed, it has been shown that NH_3_ correlate well with CO emissions after the light-off of the TWC regardless of being heavy-duty or light-duty vehicles (Giechaskiel et al., 2022a, Selleri et al., 2021). As for CO emissions, NH_3_ emissions from TWC equipped vehicles are often controlled by adjusting the air-to-fuel ratio. The presence of NH_3_ in the exhaust of engines using SCR is linked to the use of DEF. There are several physical and chemical events, reactions and process occurring in the after-treatment system during the NOx conversion process that can lead to the formation and emissions of this pollutant (for more details please see Suarez-Bertoa et al., 2020). In order to maintain NH_3_ emissions low, ammonia oxidation catalysts are applied in SCR-equipped Euro VI HD Diesel vehicles.

Both vehicles presented higher NOx emissions than those proposed in Euro 7. The Diesel bus exceeded the Cold and Hot NOx limits by 3 and 2.5 times, respectively, while the CNG exceeded them by 1.4 and 3.4 times, respectively. The results obtained during the hot tests highlight the importance of the contribution of NOx during cold start, before the catalyst reaches their operation temperature, but also after their light-off, when emissions should be very low. The CNG bus also exceeded the CO Hot Limit (by 2.4 times) and the Diesel exceeded the Hot SPN10 limit (by 1.7 times) and the two N_2_O limits (by 2.3 and 6.4 times).

N_2_O has been indicated as the most important anthropogenic Ozone Depleting Substance (ODS) (Ravishankara et al., 2009), and it is also a strong greenhouse gas with a GWP over 100 years 298 times higher than CO_2_. These high N_2_O emissions meant that the median values from the Diesel bus represented 108 gCO_2_eq/kWh and 191 gCO_2_eq/kWh for the WHVC_Cold_ and WHVC_Hot_, respectively. For the CNG, median N_2_O emissions during the WHVC_Hot_ were negligible and over the WHVC_Cold_ reached 0.044 gN_2_O/kWh. Higher values over cold start cycles compared to hot start cycles is in line with what have been reported for vehicles equipped with TWC, including CNG (Clairotte et al., 2020, Lähde and Giechaskiel, 2021). Nonetheless, in previous studies N_2_O accounted for less than 1% of the CO_2_eq (Vojtíšek-Lom et al., 2018), while the CNG bus during the WHVC_Cold_ at 35 °C reached up to 0.101 gN_2_O/kWh, or 3.5% of the CNG’s CO_2_ emissions.

**Fig. 5:**
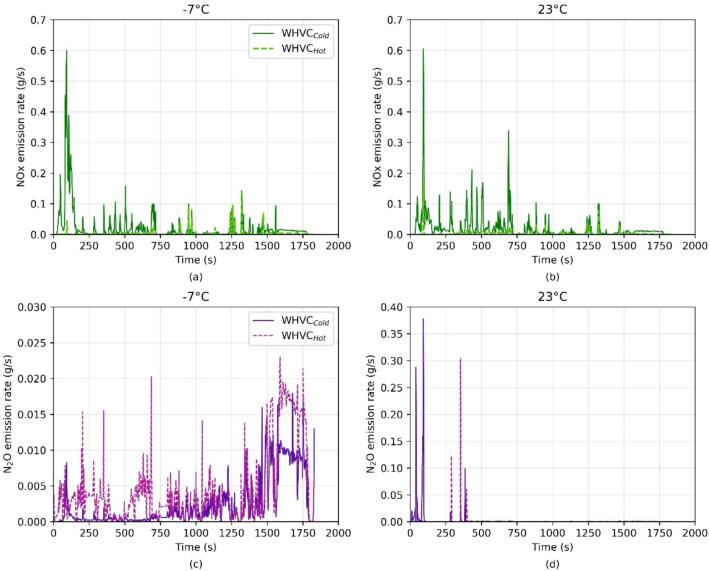
NOx (upper panels) and N_2_O (lower panels) emission rate during WHVC_Cold_ and WHVC_Hot_ at −7 °C (**a,c**) and 23 °C (**b,d**).

Like NH_3_, N_2_O also forms following different processes in the studied systems. While in the CNG it is formed on the TWC with a narrow temperature window during the catalyst light-off (Nevalainen et al., 2018), in the Diesel vehicle it can be formed through different pathways involving NOx and/or NH_3_ on the DOC, the SCR and/or the ammonia oxidation catalyst (see Preble et al., 2019, Selleri et al., 2021 for further information). As illustrated in Fig. 5 the N_2_O emissions from the Diesel bus studied were directly related to the DEF injection and SCR operation. Thus, judging the NOx emissions profiles, the DEF injection appeared to start later during the WHVC_Cold_ compared to the WHVC_Hot_, resulting in lower N_2_O emissions. This is in line with what suggested by the specialized literature (Colombo et al., 2012).

As expected, the scatter across the WHTC_Hot_ tests was very low. The trend shows that once the vehicles (engine + after-treatment/s) were at working operating temperature, the ambient temperature plays a limited role, resulting in similar emissions. During the WHVC_Cold_, the emissions of NH_3_, N_2_O and SPN10 showed high dispersion across the temperatures. The maximum values obtained during the tests showed a slightly different picture when compared to the median values. As predictable, differences with the NOx limits increased (4.3x and 2.7x for the Diesel, and 1.8x and 4.1x for the CNG, compared to the Cold and Hot limits, respectively); the CNG failed the NH_3_ Cold limit (4.9 times higher) and the SPN10 Cold limit (1.2 times higher). The Diesel bus further exceeded the N_2_O limits (2.7 and 7.2 times higher) and alongside the Hot SPN10 limit (2.0 times higher than the limit) it also failed the Cold SPN10 limit (1.2 times higher).

The highest emissions of CO, CH_4_ and NH_3_ from the CNG were observed during the tests at sub-zero temperatures. Increase of emissions of CO, CH_4_ and/or NH_3_ from TWC-equipped vehicles at sub-zero temperatures during cold start tests have been widely discussed in the literature for light-duty vehicles (Giechaskiel et al., 2022b, Suarez-Bertoa and Astorga, 2018). The increase of emissions has been explained by longer cold start periods due a longer time to reach light-off of the catalyst combined with rich operating conditions. Hence, very much in line with the results obtained for this CNG bus.

SPN23 emissions were very low, especially for the CNG vehicle. Diesel SPN23 emissions were at the expected levels, with cold start cycles having higher emissions (Napolitano et al., 2020). When looking at the SPN10 emissions, it is interesting to notice that while during WHVC_Hot_ tests the Diesel presented substantially higher emissions (up to 2 times higher than the Hot limit) than the CNG bus (maximum 15% of the Hot limit), the emissions during the WHVC_Cold_ were comparable (up to 1.2 times the SNP10 Cold limit for both Diesel and CNG, and also similar medians, 66% and 72% the limit for Diesel and CNG, respectively). Sub-23 nm particle emissions from heavy-duty Diesel vehicles are linked to urea particles emissions linked to the SCR operation (Mamakos et al., 2022, Mamakos et al., 2023). CNG heavy-duty vehicles are well known to generate particles below 23 nm possibly linked to lubrication oil (Napolitano et al., 2020). The Diesel bus, equipped with a DPF, presented consistent emissions whether the vehicle was tested hot or cold and the SPN10 emissions from CNG bus, that was not equipped with particulate filter because this was unnecessary to meet the Euro VI standard, were more affected by the cold start cold ambient tests, with the highest emissions occurring during the −7 °C tests (∼6x 10^11^ #/kWh – See Table S3 of the SM). In the case of light-duty vehicles, the impact of cold start and cold temperatures on SPN10 emissions from CNG vehicles has been linked to higher emissions (Giechaskiel et al., 2022). In case Euro 7 would have maintained the cut-off size of the measurement at 23 nm instead of reducing it to 10 nm and using the same values proposed for Euro 7 as limits, both vehicles would meet the SPN requirements.

**Fig. 6:**
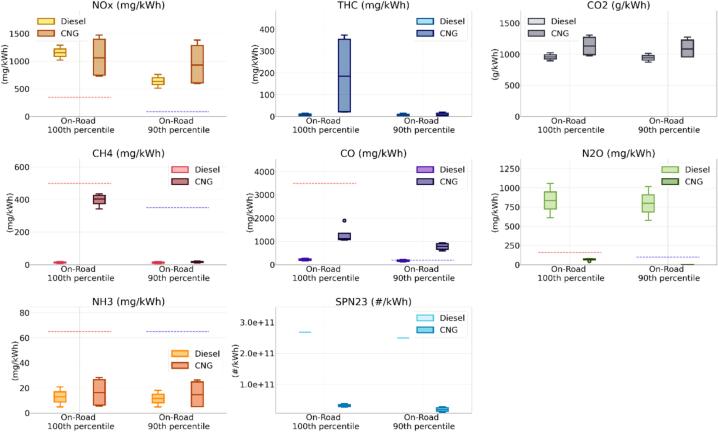
Overall emissions of the on-road tests (including ISC, ISC-like and RWT) performed in the entire testing campaign. “On-road 100th” and “On-road 90th” percentile represent the percentile of the cumulative distribution calculated using the Work-based MW approach applied to on-road tests with accumulated work higher than 3 times the reference work (i.e., WHTC work). The SPN10 measurement for both vehicles was not available for these tests. No correction has been applied for any of the instruments used. Box plot are Q1-Q3 interquartiles, horizontal line Q2. The whiskers extend to 1.5 times the Q3-Q1 difference. Dots indicate outliers. Dotted red and blue lines indicate the Euro 7 limits, as laid down in the European Commissions’ proposal, respectively for 100^th^ percentile and 90^th^ percentile emissions. (For interpretation of the references to colour in this figure legend, the reader is referred to the web version of this article.)

Fig. 6 illustrates the results got from the on-road and ISC-like tests analyzed according to what described in the Section 2.4.2 for tests that accumulated over 3 times the reference work (i.e., 76.4 and 75.9 kWh, respectively for the Diesel and CNG). Similar to what obtained for the WHVC_Cold_ tests, the CNG’s worst window (i.e., the 100^th^ percentile window) presented higher emissions than Diesel for THC, CH_4_ and CO. The emissions of the worst windows from Diesel were higher than the CNG for N_2_O and SPN23, as they were for the WHVC tests (SPN10 measurement was not available for the on-road and ISC-like test).

The pollutants surpassing the limits on-road were the same as those exceeding the WHVC. The level of these exceedances, however, changed, increasing substantially – See Table S3 in the Supplementary Material. Hence, the CNG exceeded the 90^th^ percentile limit of CO by 4 times. The Diesel exceeded the 100^th^ and 90^th^ percentile limit of N_2_O by 5.2 and 8 times, respectively. This means that the median of 100^th^ and 90^th^ percentile windows were 0.837 and 0.799 gN_2_O/kWh, with a maximum at 1.061 gN_2_O/kWh (i.e., up to 37% of the CO_2_ emitted by this vehicle during the WHVC). High N_2_O emissions, reaching up to 0.196 g/kWh, have been previously reported for Euro VI HDV (Giechaskiel et al., 2019, Mendoza-Villafuerte et al., 2017, Selleri et al., 2022). Note that US EPA requires that HD do not emit over 0.133 g/kWh of N_2_O. As stated above, N_2_O emissions from vehicles equipped with TWC have been considered negligible (Clairotte et al., 2020) and the emissions of this CNG bus during the WHVC were indeed close to zero during the hot tests and minor during the cold ones. However, unlike the WHVC_Cold_ tests, there were emissions of N_2_O in the 100^th^ percentile windows during the on-road and ISC-like tests regardless the ambient temperature. The emissions ranged from 0.068 gN_2_O/kWh to 0.080 gN_2_O/kWh and, as for the WHVC_Cold_ tests, they were concentrated on the cold start, with low emissions in 90^th^ percentile windows (up to 0.003 gN_2_O/kWh).

**Fig. 7:**
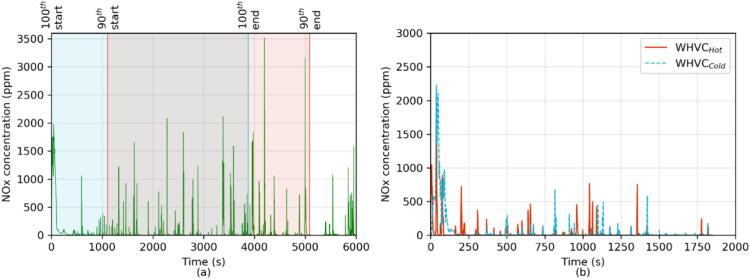
CNG bus NOx concentration (ppm) at tailpipe for: **a)** (left panel) on-road (green solid line) test, where blue shaded area indicates the 100^th^ percentile window and the red shaded area the 90^th^ percentile window. To indicate where the window starts and finish, the 100^th^ percentile window length (blue shaded area) goes from the indication 100^th^ start to 100^th^ end, and the 90^th^ percentile window length (red shared area) from 90^th^ start to 90^th^ end. b) (right panel) WHVC_Cold_ (red solid line) and WHVC_Hot_ (blue dashed line). Note that, for illustration purposes, the on-road test timescale was limited only to 6000 s. b) (right panel) WHVC_Cold_ (red solid line) and WHVC_Hot_ (blue dashed line). (For interpretation of the references to colour in this figure legend, the reader is referred to the web version of this article.)

Median NOx emissions on-road for the worst window of the Diesel (1.155 g/kWh) bus were comparable to those obtained during the WHVC_Cold_, showing a common effect of the emissions during cold start. However, in the CNG case the median emissions of the worst on-road windows (1.062 g/kWh) were 2.2 times higher than those got over the WHVC_Cold._ Moreover, in the case of the 90^th^ percentile window the CNG vehicle presented similar emissions (0.932 g/kWh) to those of the worst window consistently with the high NOx emissions measured all along the test. Unlike during the WHVC tests, where NOx was 36% lower during WHVC_Hot_ as compared to WHVC_Cold_, during the on-road and ISC-like tests the 100^th^ percentile window, which included the cold start, were 14% higher than the 90^th^ percentile window, which started well after the light-off of the TWC. Fig. 7 shows the presence of further NOx emissions peaks from the CNG vehicle even after the cold start phase. As consequence, the CNG exceeded the 90^th^ percentile limit by 10 times. After the cold start, the Diesel’s emissions improved (0.639 g/kWh), remaining however still high, i.e., 7.1 times the limit.

Comparing the emission factors obtained for the 90^th^ percentile window of the ISC_I_ and ISC_III_ tests using the Euro 7 methodology (e.g., NOx Diesel 0.763 g/kWh and CNG 0.605 g/kWh) and using the Euro VI-D, highlights that the emissions excluded by previous procedures led to 48% and 57% lower emission factors. Moreover, the NOx emission factors obtained using all the emissions, including cold start, are about 2 times higher than those reported by Zhu et al. (2024), using EPA’s and CARB’s 2/3 BIN MAW methodologies.

On-road and ISC-like NH_3_ emissions of the CNG bus were about on order of magnitude lower compare to those reported by Zhu et al. (2024) and to Thiruvengadam et al. (2016), which reported 0.10–0.19 gNH_3_/bhp-hr (0.134 – 0.255 gNH_3_/kWh). They were similar in the case of Diesel. These emissions were below the Euro 7 limit for both vehicles and similar to those achieved in the laboratory during tests with comparable ambient conditions. All this suggests that, although the NH_3_ emissions remain high, they are lower than in USA where it is not regulated.

## 4 Conclusions

Two Heavy-Duty Euro VI Step D buses, one powered by a Diesel engine and the other one by a spark ignition engine fueled running on cCNG, were tested on road and on a chassis dyno at different ambient temperatures. Gaseous emissions (including NOx, THC, CH_4_, CO, N_2_O and NH_3_) and SPN (SPN10 and SPN23) were measured using PEMS and laboratory grade equipment. The emission data from the two vehicles were analyzed according to Euro VI D regulatory framework and according to the Commission's proposed provisions for the Euro 7.

The two vehicles studied meet the Euro VI D standard for which they were designed. These included on-road ISC tests and tests on dyno using the WHVC, which were used as a proxy of the engine type approval cycle WHTC.

The main differences in the emissions between the Diesel and the CNG vehicles were higher N_2_O and SPN23 emissions from the Diesel vehicle compared to the CNG and higher NH_3_, CH_4_ and CO emissions from the CNG vehicle. NOx emissions from the Diesel vehicle were higher than those of the CNG during the WHVC_Cold_ tests, but comparable, or slightly lower, during the on-road tests and WHVC_Hot_.

The technologies and strategies used in these two vehicles would need to be improved substantially to comply with the Euro 7 proposal made by the European Commission. When analyzed from the Euro 7 proposal perspective, the CNG vehicle exceeded the limits for NOx, CH_4_, CO, NH_3_ and SPN10 and the Diesel exceeded the limits for NOx, N_2_O and SPN10. Even though the ambient conditions were milder during the on-road tests, the emissions were higher than those measured during the WHVC tests.

High emissions of CO, CH_4_, NH_3_ and SPN10 were measured from the CNG, especially at −7 °C. Interestingly, N_2_O emissions from this vehicle were not negligible, reaching up 0.101 g/kWh or 30 gCO_2_eq/kWh (3% of the vehicle’s CO_2_). The contribution of CH_4_ in terms of CO_2_eq was up to 40 g/kWh (4.6% of the vehicle’s CO_2_).

The Diesel presented extremely high emissions of N_2_O, reaching up to 1.061 gN_2_O/kWh or in other terms, 316 gCO_2_eqv/kWh, i.e., ∼37% of the CO_2_ emitted this vehicle during the WHVC. Given the extremely high values recorded, the potential contribution of road transport to the N_2_O budget should not be neglected, especially since this pollutant is an important ozone depleting substance. Moreover, the different contribution of each pollutant resulting from the two technologies, underlines the importance of enforcing individual limits for them.

Although maximum NH_3_ emissions factors were lower than those extremely high (up to 0.320 g/kWh), measured from the CNG, the emissions from the Diesel (0.021 g/kWh) were not negligible and comparable to those typically reported for modern heavy-duties equipped with ammonia oxidation catalyst.

When considering all emissions and not just those required to considered by the Euro VI D standard (important to note that this has been superseded by the Euro VI E), NOx emissions from both vehicles were high. Besides the high emissions during cold start, that were not regulated for this Euro VI D vehicles, emissions remained high also during the rest of the test.

Both vehicles complied with the 23 nm SPN limits. However, it was observed that meeting future SPN10 limits for cold start tests can be challenging. The high sub-23 nm levels, typically linked to SCR-formed urea particles for Diesel vehicles, and lubrication oil for CNG vehicles, led up to 2 times exceedance of the proposed Euro 7 limits.

## Funding sources

This research did not receive any specific grant from funding agencies in the public, commercial, or not-for-profit sectors.

## CRediT authorship contribution statement

**Roberto Gioria:** Writing – original draft, Methodology, Investigation, Formal analysis, Data curation, Conceptualization. **Tommaso Selleri:** Writing – review & editing, Investigation, Conceptualization. **Barouch Giechaskiel:** Writing – review & editing, Methodology, Investigation, Data curation. **Jacopo Franzetti:** Writing – review & editing, Visualization, Investigation. **Christian Ferrarese:** Writing – review & editing, Visualization, Investigation, Formal analysis, Data curation. **Anastasios Melas:** Writing – review & editing, Investigation, Data curation. **Fabrizio Forloni:** Writing – review & editing, Investigation. **Ricardo Suarez-Bertoa:** Writing – review & editing, Writing – original draft, Supervision, Formal analysis, Conceptualization. **Adolfo Perujo:** Writing – review & editing, Project administration, Conceptualization.

## Declaration of competing interest

The authors declare that they have no known competing financial interests or personal relationships that could have appeared to influence the work reported in this paper.
