# Supplementary material for: Regulated and unregulated emissions from Euro VI Diesel and CNG heavy-duty vehicles
Source: Transp Res D Transp Environ. 2024 Sep;134:104349. doi: 10.1016/j.trd.2024.104349 (PMC11367066; doi:10.1016/j.trd.2024.104349)
Supplement: Supplementary Data 1 [file mmc1.docx]

**Regulated and unregulated emissions from Euro VI Diesel and CNG heavy-duty vehicles**

**Roberto Gioria^1*^, Tommaso Selleri^1,2^, Barouch Giechaskiel^1^, Jacopo Franzetti^1,3^, Christian Ferrarese^1,3^, Anastasios Melas^1^, Fabrizio Forloni^1^, Ricardo Suarez-Bertoa^1*^, and Adolfo Perujo^1^**

^1^ European Commission, Joint Research Centre (JRC), 21027 Ispra, Italy.

^2^ European Environment Agency (EEA), 1050 Copenhagen, Denmark.

^3^ ETSI Minas y Energía, Universidad Politécnica de Madrid, Paseo Juan XXIII 11, Madrid, Spain

* Correspondence: [roberto.gioria@ec.europa.eu](mailto:roberto.gioria@ec.europa.eu) and ricardo.suarez-bertoa@ec.europa.eu

**Figure S1**. On road test measurement setup. (EFM = Exhaust Flow Meter; GPS = Global Positioning System; PEMS AVL MOVE = Portable Emission Measurement System manufactured by AVL; OBS-ONE-XL = HORIBA’s Infrared Laser Absorption Modulation (IRLAM) analyser; PEMS-LAB = portable FTIR analyser (p-FTIR) by CERTAM-ADDAIR.


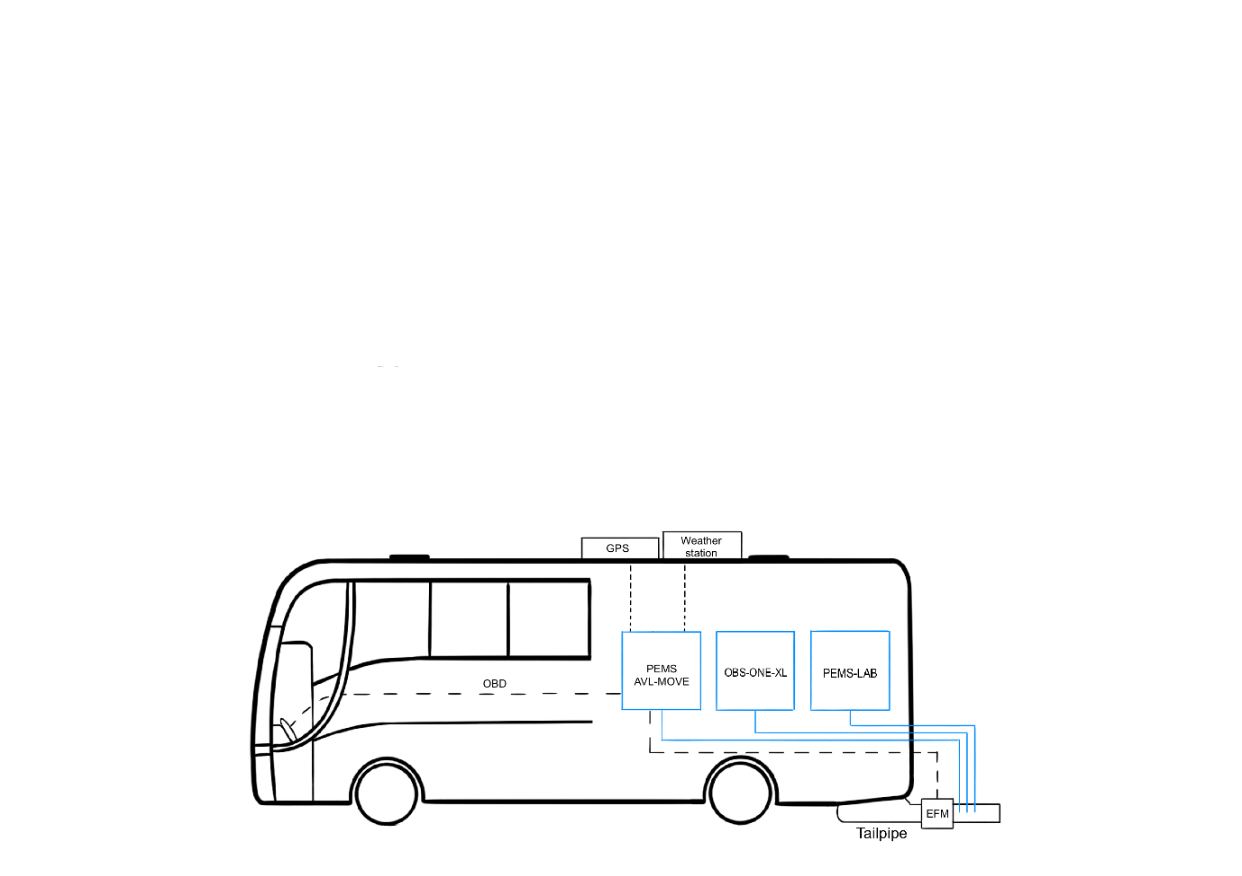


**Figure S2.** Trip segmentation as required by Regulation(EU) 2016/1718 for the two vehicle categories under test. **a)** In–Service Conformity test M3 Class III – Diesel bus (nominal values U: 45%; R: 25%; M: 30%). **b)** In–Service Conformity test M3 Class I – CNG bus (nominal values U: 70%; R: 30%). Figure a) shows just one example for the Diesel ISC route (in blue) and is directly compared to a RWT route for CNG (in red), demonstrating the equivalence of the two tests. While in Figure b) are illustrated both ISC routes used for CNG assessment. Upper and lower limits (dashed black lines) indicate the allowable range percentage share in time, considering the nominal value and a regulated tolerance of ±5%. All the RWTs have been analyzed using the speed base method, specific of the previous regulatory steps (Step A-C). Route's statistics based on the vehicle speed are summarized in Table S1.

**
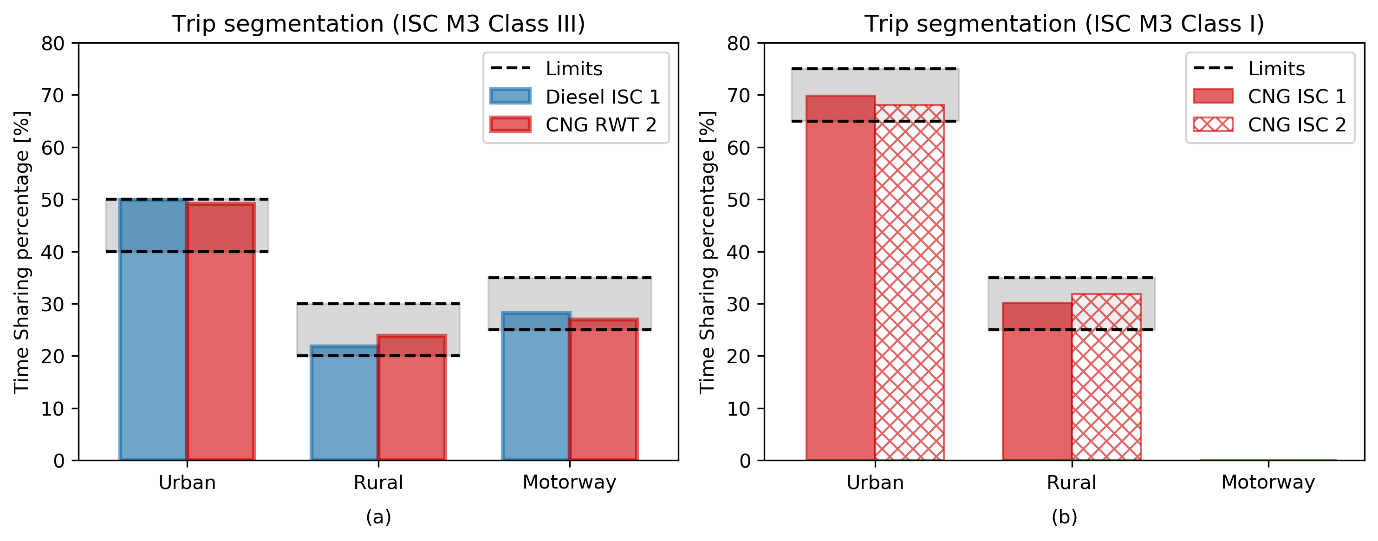
**

**Figure S3**. **a)** WHVC for CNG bus (max speed limited at 85 km/h). **b)** ISC_III_-like for Diesel bus (max speed limited at 100 km/h). The vehicle speed is reported in blue; the exhaust temperature in red. These graphs are shown as an example of possible combinations of laboratory tests.

**
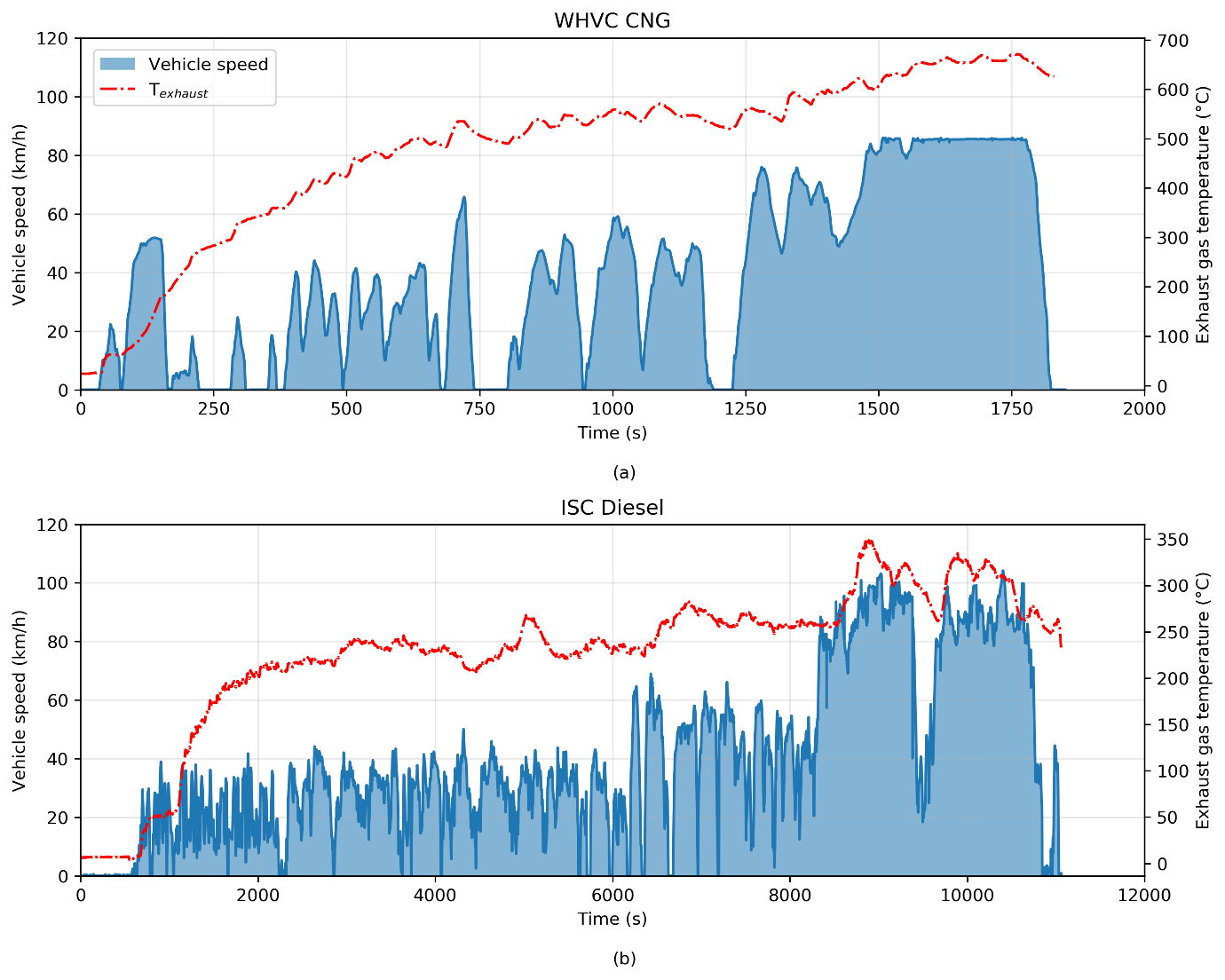
**

**Figure S4.** Overview of the equipment used during the laboratory experiments


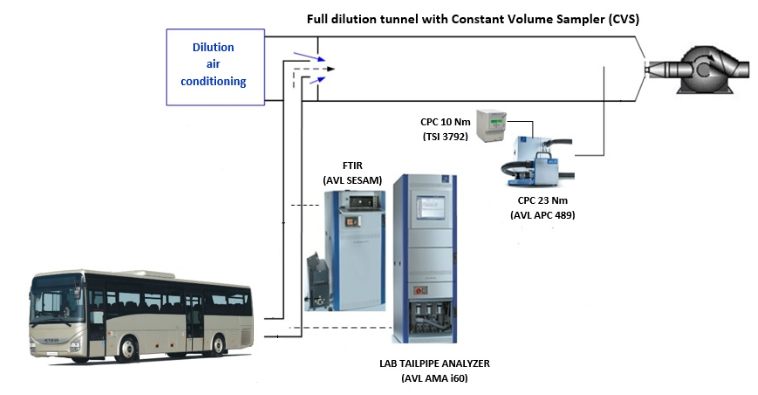


**Figure S5.** Tailpipe exhaust gas temperature for Diesel (dashed lines) and CNG (continuous lines) vehicles for every road test.


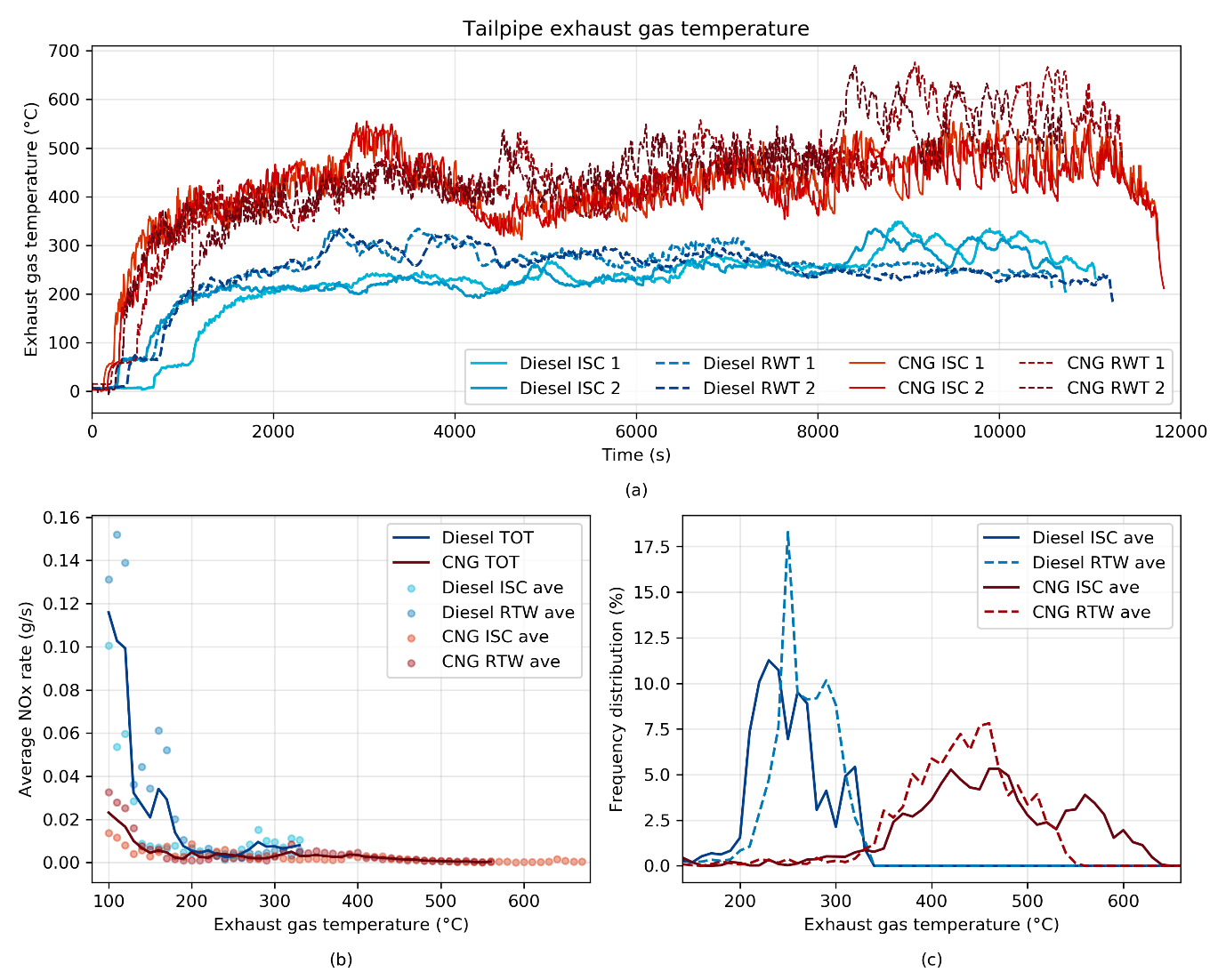


**Table S1.** Routes statistics based on the vehicle speed according to first acceleration method (FAM) for the in-service conformity (ISC) and speed based method for the RWT route.

|  |  |  |  |  |  |  |  |  | **Evaluation using FAM method** | | |
| --- | --- | --- | --- | --- | --- | --- | --- | --- | --- | --- | --- |
| **Bus by fuel type** | **Test Cycle** | **Trip**  **Duration (min)** | **Trip Distance (km)** | **Trip**  **Work**  **total**  **(kWh)** | **Aver.**  **Speed  (km/h)** | **Aver. Amb.**  **Temp.**  **(K)** | **Aver. Rel. Humidity (%)** | **Aver.**  **Altitude**  **(m)** | **Urban**  **Distance**  **(km)** | **Rural**  **Distance**  **(km)** | **Motorway**  **Distance**  **(km)** |
| Diesel | Diesel ISC_1 | 175 | 121 | 117.8 | 41.6 | 279 | 72 | 244 | 38.9 | 24.7 | 57.8 |
|  | Diesel ISC_2 | 176 | 121 | 123.5 | 41.2 | 276 | 98 | 245 | 38.8 | 24.9 | 57.3 |
|  | Diesel RTW_1 | 175 | 147 | 136.8 | 50.4 | 278 | 90.0 | 247 | 5.4^2^ | 10.6^1^ | 131.1^1^ |
|  | Diesel RTW_2 | 184 | 147 | 134.5 | 47.9 | 279 | 95 | 247 | 5.4^2^ | 10.6^1^ | 131.2^1^ |
| CNG | CNG RTW_1 | 186 | 122 | 126.1 | 39.3 | 279 | 55 | 249 | 38.4 | 26.3 | 57.3 |
|  | CNG RTW_2 | 175 | 121 | 111.6 | 41.5 | 278 | 69 | 247 | 41.3 | 25.7 | 54.1 |
|  | CNG ISC_1 | 194 | 100 | 112.8 | 31.0 | 277 | 94 | 260 | 59.7 | 40.6 | - |
|  | CNG ISC_2 | 195 | 100 | 105.5 | 30.8 | 278 | 91 | 259 | 59.5 | 40.7 | - |

^1^FAM not applicable

**Table S2:** Summary for on-road tests of all pollutants, where the measurements are reported in mg/kWh, g/kWh or #/kWh. Median, maximum and minimum values are reported for the Diesel and the CNG vehicle.

| Pollutant | Fuel | Percentiles | Median | Max | Min |
| --- | --- | --- | --- | --- | --- |
| NOx (mg/kWh) | Diesel | 100^th^ | 1155 | 1291 | 1019 |
|  |  | 90^th^ | 639 | 763 | 515 |
|  | CNG | 100^th^ | 1062 | 1473 | 731 |
|  |  | 90^th^ | 932 | 1384 | 597 |
| THC (mg/kWh) | Diesel | 100^th^ | 8 | 15 | < 1 |
|  |  | 90^th^ | 8 | 15 | < 1 |
|  | CNG | 100^th^ | 185 | 374 | 21 |
|  |  | 90^th^ | 7 | 20 | < 1 |
| CO_2_ (g/kWh) | Diesel | 100^th^ | 960 | 1026 | 895 |
|  |  | 90^th^ | 945 | 1014 | 875 |
|  | CNG | 100^th^ | 1132 | 1310 | 976 |
|  |  | 90^th^ | 1088 | 1279 | 957 |
| CH_4_ (mg/kWh) | Diesel | 100^th^ | 13 | 19 | 7 |
|  |  | 90^th^ | 13 | 19 | 7 |
|  | CNG | 100^th^ | 404 | 435 | 343 |
|  |  | 90^th^ | 19 | 24 | 12 |
| CO (mg/kWh) | Diesel | 100^th^ | 224 | 283 | 166 |
|  |  | 90^th^ | 176 | 236 | 117 |
|  | CNG | 100^th^ | 1127 | 1357 | 1069 |
|  |  | 90^th^ | 794 | 942 | 605 |
| N_2_O (mg/kWh) | Diesel | 100^th^ | 837 | 1061 | 613 |
|  |  | 90^th^ | 799 | 1021 | 577 |
|  | CNG | 100^th^ | 75 | 80 | 68 |
|  |  | 90^th^ | < 1 | 3 | < 1 |
| NH3  (mg/kWh) | Diesel | 100^th^ | 13 | 21 | 5 |
|  |  | 90^th^ | 12 | 18 | 5 |
|  | CNG | 100^th^ | 16 | 29 | 6 |
|  |  | 90^th^ | 15 | 26 | 5 |
| SPN23 (#/kWh) | Diesel* | 100^th^ | 2.7e11 | 2.7e11 | 2.7e11 |
|  |  | 90^th^ | 2.5e11 | 2.5e11 | 2.5e11 |
|  | CNG | 100^th^ | 3.2e10 | 3.8e10 | 2.6e10 |
|  |  | 90^th^ | 1.9e10 | 2.9e10 | 9.9e09 |

*Max and Min values do not consider outliers.*

**Only one on-road test with Diesel fuel was performed measuring SPN23*

**Table S3: WHVC.** Comprehensive summary for WHVC Lab tests of all pollutants, where the measurements are reported in mg/kWh, g/kWh or #/kWh. Median, maximum and minimum values are reported for every test case and vehicle (Diesel or CNG) analysed. All tests at each ambient temperature are reported.

| Pollutant | Fuel | WHVC Cold/Hot | Median | Max | Min |
| --- | --- | --- | --- | --- | --- |
| NOx (mg/kWh) | Diesel | WHVC Cold | 1035 | 1516 | 805 |
|  |  | WHVC Hot | 222 | 247 | 174 |
|  | CNG | WHVC Cold | 478 | 644 | 342 |
|  |  | WHVC Hot | 306 | 371 | 243 |
| THC (mg/kWh) | Diesel | WHVC Cold | 7 | 8 | 5 |
|  |  | WHVC Hot | 4 | 5 | 3 |
|  | CNG | WHVC Cold | 405 | 480 | 295 |
|  |  | WHVC Hot | 17 | 23 | 10 |
| CO_2_ (g/kWh) | Diesel | WHVC Cold | 803 | 809 | 787 |
|  |  | WHVC Hot | 789 | 798 | 768 |
|  | CNG | WHVC Cold | 856 | 927 | 830 |
|  |  | WHVC Hot | 815 | 872 | 770 |
| CH_4_ (mg/kWh) | Diesel | WHVC Cold | 2 | 3 | 2 |
|  |  | WHVC Hot | 2 | 3 | 1 |
|  | CNG | WHVC Cold | 388 | 460 | 284 |
|  |  | WHVC Hot | 17 | 21 | 6 |
| CO (mg/kWh) | Diesel | WHVC Cold | 562 | 2368 | 106 |
|  |  | WHVC Hot | 111 | 174 | 76 |
|  | CNG | WHVC Cold | 1805 | 3083 | 852 |
|  |  | WHVC Hot | 472 | 688 | 304 |
| N_2_O (mg/kWh) | Diesel | WHVC Cold | 363 | 426 | 324 |
|  |  | WHVC Hot | 642 | 716 | 593 |
|  | CNG | WHVC Cold | 44 | 101 | 9 |
|  |  | WHVC Hot | < 1 | < 1 | < 1 |
| NH3  (mg/kWh) | Diesel | WHVC Cold | 1 | 1 | < 1 |
|  |  | WHVC Hot | < 1 | 1 | < 1 |
|  | CNG | WHVC Cold | 34 | 320 | 17 |
|  |  | WHVC Hot | 18 | 26 | 11 |
| SPN23 (#/kWh) | Diesel | WHVC Cold | 1.2e11 | 5.0e11 | 8.5e10 |
|  |  | WHVC Hot | 1.2e11 | 1.5e11 | 8.4e10 |
|  | CNG | WHVC Cold | 8.6e10 | 1.8e11 | 9.1e09 |
|  |  | WHVC Hot | 8.7e09 | 1.6e10 | 6.1e09 |
| SPN10 (#/kWh) | Diesel | WHVC Cold | 3.3e11 | 6.0e11 | 2.5e11 |
|  |  | WHVC Hot | 3.4e11 | 3.9e11 | 3.1e11 |
|  | CNG | WHVC Cold | 3.6e11 | 6.2e11 | 4.0e10 |
|  |  | WHVC Hot | 2.2e10 | 3.0e10 | 1.4e10 |
| HCHO (mg/kWh) | Diesel | WHVC Cold | < 1 | < 1 | < 1 |
|  |  | WHVC Hot | < 1 | < 1 | < 1 |
|  | CNG | WHVC Cold | < 1 | 2 | < 1 |
|  |  | WHVC Hot | < 1 | < 1 | < 1 |

*Max and Min values do not consider outliers.*

|  |  |
| --- | --- |
